# Antiphospholipid antibody syndrome with thrombotic splenic infarcts associated with acute cytomegalovirus infection

**DOI:** 10.1099/acmi.0.000032

**Published:** 2019-06-10

**Authors:** Christopher Denham, Ginger Tissier, Amit Golding

**Affiliations:** ^1^ Trinity School of Medicine, Kingstown, St.Vincent and the Grenadines, West Indies; ^2^ Baltimore VA/VAMHCS and University of Maryland School of Medicine, Baltimore, MD, USA

**Keywords:** acute CMV infection, thrombosis, antiphospholipid antibodies, anti-coagulation, antiphospholipid antibody syndrome

## Abstract

**Introduction:**

We describe a case of acute cytomegalovirus (CMV) infection complicated by acquired antiphospholipid antibodies and splenic thrombi. We discuss the associations between CMV infection and thrombosis risk and correlation with antiphospolipid antibodies.

**Case presentation.:**

A previously healthy 32-year-old woman is hospitalized for acute abdominal pain and fever and found to have multiple splenic infarcts on an abdominal computed tomography (CT) scan. An infectious work-up is negative except for acute CMV, and a hypercoagulable work-up is only positive for antiphospholipid antibodies. The patient is discharged and placed on anti-coagulation therapy for 6 months.

**Conclusion:**

Co-incident thrombosis and antiphospholipid antibody syndrome can occur with acute viral infections, including CMV. We discuss the viral infection-associated increased risk of developing blood clots and antiphospholipid antibodies as being either correlative with or causative of viral-induced thrombosis.

## Introduction

Viral infections can stimulate the transient development of autoimmunity and thrombosis in certain individuals. Primary cytomegalovirus (CMV) infection in healthy individuals can be asymptomatic or cause mononucleosis-like symptoms. Recent case reports provide evidence for CMV being the causal factor inducing antiphospholipid antibody syndrome (APS) with associated vascular thrombosis. APS is defined by arterial or venous thromboses and recurrent foetal losses. APS is confirmed by the presence of anti-cardiolipin antibodies, lupus anticoagulant, or anti-*β*2 glycoprotein I (*β*2-GPI) antibodies [1]. We describe a patient who developed primary CMV infection with subsequent splenic thrombosis associated with a transient rise in antiphospholipid (aPL) antibodies. We discuss the role acute CMV infection and aPL antibodies play in the development of subsequent thrombosis.

## Case

A healthy 32-year-old female with no history of thrombosis or livedo reticularis was in good health until the last week of September 2017. At that time, she began to develop epigastric pain with tenderness that radiated to the lower abdominal region. Over the next 11 days the patient developed bilateral wrist and knee joint pain and stiffness, recurrent daily fevers, nausea and vomiting, profuse diaphoresis and a lacy rash throughout her body. The patient sought medical treatment and was admitted to a local hospital.

A physical examination on admission revealed a temperature of 39.2 °C, a pulse of 123, blood pressure of 129/81, respirations of 20 and oxygen saturation of 98 % on room air. Her skin was diffusely erythematous with a lacy rash. An abdominal examination revealed guarding and pain out of proportion to light palpation in all quadrants, with this being most pronounced in the epigastric region.

Laboratory studies performed on admission showed a white blood cell count of 6.3/mm³. Hematocrit and haemoglobin were 11.2 g/dL and 33%, respectively. ESR was 8 mm/h^−^^1^ and CRP was 1.7 mg/dL. Liver function studies revealed an elevated aspartate aminotransferase of 446 (normal: 14–36 units/L), elevated alanine aminotransferase of 839 (normal: 9–52 units/L), elevated lactate of 2.6 (normal: 0.5–2 mmol/L) and total bilirubin of 0.9 mg/dL.

**Fig. 1.**
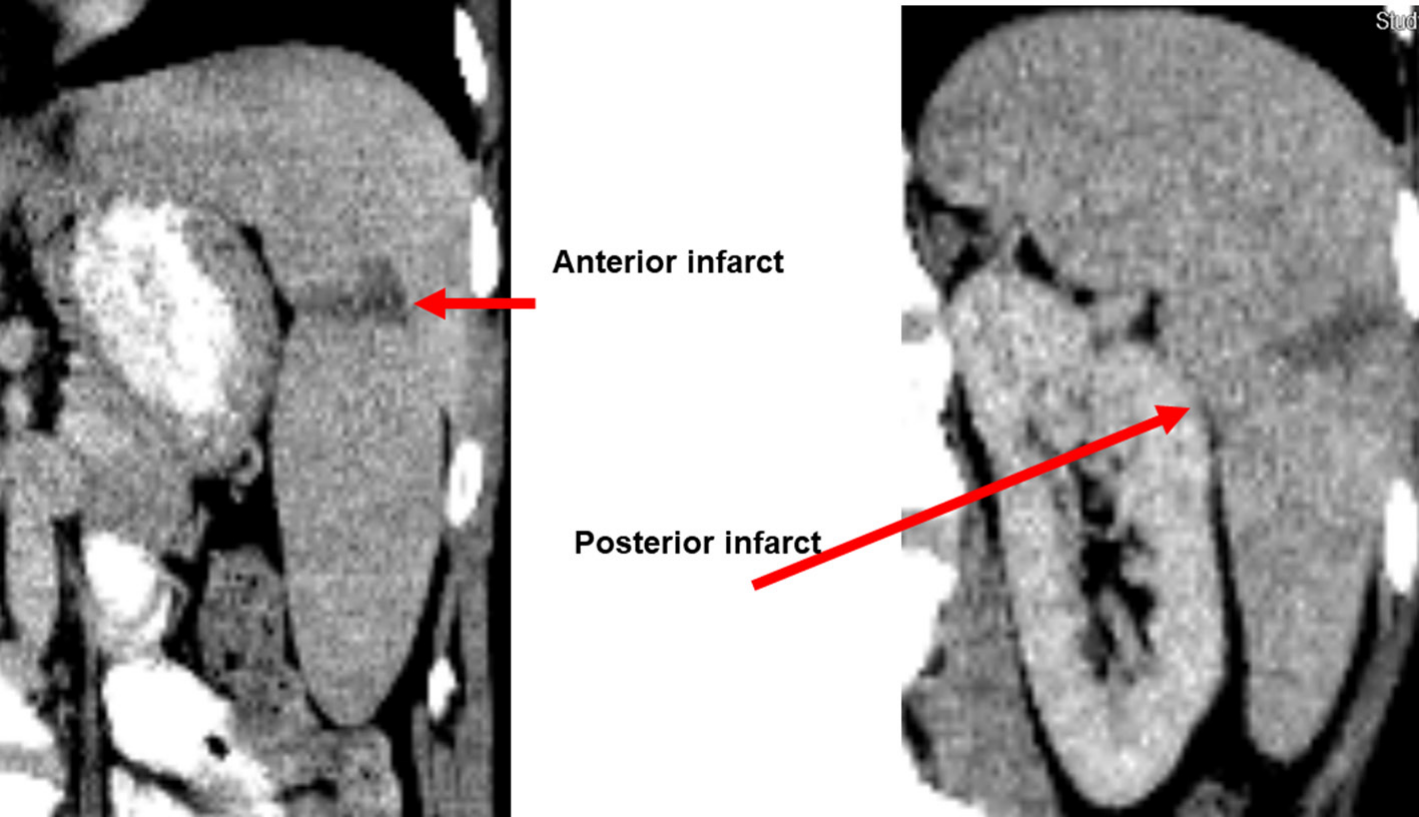
Contrast-enhanced CT scan of the abdomen showing evidence of splenic infarcts. (a) mild splenomegaly with wedge-shaped infarct in the anterior portion of the mid upper spleen; stable. (b) there is a similar wedge-shaped area of infarct in the posterior aspect of the spleen at the same level.

A contrast-enhanced computed tomography (CT) scan of the abdomen and pelvis revealed hepatosplenomegaly, multiple wedge-shaped anterior and posterior splenic infarcts coursing from the lateral aspect of the capsule to the splenic hilar region (Fig. 1), and moderate thickening of the descending and transverse colonic wall, suggestive of colitis. A pelvic ultrasound revealed moderate free fluid. A CT angiogram revealed no vasculitis or vascular insufficiency. A transesophageal echocardiogram revealed no vegetations or valvular insufficiency. Magnetic resonance imaging (MRI) of the brain was performed because of waxing/waning mental status; the results were unremarkable.

The work-up for hypercoagulability revealed a positive lupus anticoagulant with a partial thromboplastin time of 35.8 (normal: 25–35 s) and a PTT/LA mixing study time of 52 (normal: <40 s); a thromboplastin time of 11.5 (normal 10–12 s) , an antithrombin III level of 126 (normal: 80–120 %), a protein C level of 121 (normal: 70–180 %) and a protein S level of 29 (normal: 60–140 %). A number of tests were performed to detect APS antibodies: lupus anticoagulant (11.4; normal, <11.5 seconds), beta 2 glycoprotein I Ab IgG/A/M (9; normal, <20), anticardiolipin antibody IgG (<14; normal, <14); anticardiolipin IgM (>150; normal, <12) IgA (<11; normal, <11). There was also positive detection of anti-phosphotidyl serine (APSA) IgM. A formal haematology consultation was requested. Their documented conclusion was that positive anticardiolipin IgM and lupus anticoagulant with splenic infarcts could point to a diagnosis of antiphospholipid antibody syndrome justifying anti-coagulation therapy, but would require follow-up positive APS antibody testing for confirmation. Follow-up testing for ACA and APSA antibodies remained positive up to 7 months following the initial presentation and after anti-coagulation therapy was completed (see Table 1).

**Table 1.** Changes in antiphospholipid antibody titres and CMV antibody and DNA levels over time

Patient titre results			
	10/16	01/17	05/17
ACA IgM	**>** **150**	**23**	<9
APSA IgM	**>** **100**	**45**	**31**
CMV IgM	**>** **240**		
CMV DNA log 10	**3.2**	<200	Undetectable

ACA, anti-cardiolipin antibody; APSA, anti-phosphatidylserine antibody.

Blood and urine cultures were performed to determine the aetiology of the fever of unknown origin, and the results were negative. The infectious serologies and DNA tests for multiple viral infections were negative for the presence of HIV, hepatitis A, hepatitis B, hepatitis C and Epstein–Barr virus. The serology for other infections, including malaria, Lyme, *Ehrlichiosis*, Q fever, *Rickettsia *, West Nile, *Bartonella *, *Histoplasma* and *Aspergillus*, was also negative. Serological tests for rheumatological conditions, including ANA, anti-SS-A/SS-B, actin smooth muscle Ab, SM/RNP Ab and anti-CCP, were also negative. However, testing revealed the presence of a primary infection of CMV (which could have been due to either endogenous reactivation or exogenous reinfection): the CMV IgM titre was 240 AU ml^−1^, IgG was positive and CMV DNA was 1598 IU ml^−1^ during the first week of admission (Table 1).

Initially, the patient had been treated for suspected Lyme, *ehrlichiosis* and *babesiosis* infection with doxycycline and ceftriaxone, with vancomycin being started on day two of admission. Vancomycin was discontinued due to red man syndrome, at which point a rapid response was initiated and the patient was given IV benadryl, which ameliorated the episode. After a CT scan of the abdomen showed evidence for multiple splenic infarcts, coagulation studies confirmed the presence of antiphospholipid antibodies and prophylactic IV heparin was initiated, followed by lovenox at discharge. The patient was discharged 11 days after admission in fair condition. Several months after discharge, the patient had multiple near-syncopal episodes with tachycardia, orthostatic hypotension and diaphoresis. A tilt table test was performed, which confirmed the diagnosis of postural orthostatic tachycardia syndrome (POTS). The patient was treated with ivabradine, which provided no relief and exacerbated her symptoms. Treatment with compression stockings and sodium chloride 1 g tablets provided moderate, sporadic relief. Episodes of POTS continue to trouble the patient, although they have decreased in frequency. Five months after discharge, CMV DNA became undetectable and the titres of IgM and IgG came down, suggesting resolution of the primary infection (Table 1). The patient discontinued lovenox shortly thereafter. The patient continues to be free of symptoms of hypercoagulability, while intermittent episodes of livedo reticularis persist.

## Discussion

The prevalence of CMV infections in the general population ranges from 50–80 % in developed countries such as the USA and Europe, while worldwide prevalence is estimated to be 60–100 % in developing countries such as Africa and Asia [2]. Prevalence also increases with age. In all individuals, primary infection is followed by latency, which can lead to reactivation during life. Primary CMV infection is diagnosed with CMV IgM and CMV DNA detection, while positive CMV IgG titres are confirmatory for a primary infectious process. An acute/primary CMV infection can result from either endogenous reactivation or exogenous reinfection and is typically rather innocuous, with little impact on immunocompetent individuals. Most healthy patients will have mononucleosis-like symptoms for several days, while others will be entirely asymptomatic. However, in immunocompromised individuals and foetuses, infection can have devastating outcomes [3–5]. In some patients, infection can lead to thrombocytopenia, haemolytic anaemia, meningoencephalitis, myocarditis, retinitis, interstitial pneumonitis, prolonged fever and hepatitis. While the original reports of thrombosis primarily concerned immunocompromised patients, more recent data have revealed a correlation between primary CMV infection and thrombotic events in immunocompetent patients [6].

In a recent meta-analysis of thrombosis associated with primary CMV infection, 66 % of patients were immunocompetent and 20 % of those patients developed APS [7]. Predisposing risk factors (protein C and S deficiency, factor V leiden mutation) have been identified in a few case reports, while CMV infection has been noted to be the primary cause in most cases [8–10]. Several mechanisms have been elucidated for CMV-induced thrombosis. It can cause vascular damage that activates coagulation factors and adhesion of platelets and leukocytes [11–14]. Furthermore, CMV can cause thrombophilia via promotion of factor VIII or thrombin while limiting production of heparin sulfate, further inhibiting anti-coagulation pathways [9]. CMV can also trigger the production of antiphospholipid antibodies. Although the mechanism remains unknown, molecular mimicry has been proposed in one theory [15–17].

The presence of antiphospholipid antibodies is associated with APS. It presents as venous or arterial thrombosis, thrombocytopenia and recurrent pregnancy loss. It is common for arterial manifestations to include cerebrovascular infarction, whereas lower limb venous thrombosis and pulmonary embolisms are significant in the venous system [1, 18]. However, insults can occur anywhere throughout the vascular system. Antiphospholipid antibodies occur in two forms: the first is associated with primary or secondary antiphospholipid syndrome [18] and the second is associated with the elderly [19], infections [20, 21] and drugs. Antiphospholipid antibodies are associated with infectious diseases, such as syphilis [22], hepatitis C virus, CMV, varicella zoster virus, adenovirus, parvovirus, rubella virus, mumps virus [20, 21] and *Borrelia burgdorferi* [23], and *Plasmodium* species, *Schistosoma* species and *Mycobacterium tuberculosis* [24]. It is significant to note that the presence of these antibodies in the aforementioned pathogens is not usually associated with APS. APS associated with infection is often considered to be non-pathogenic and non-thrombogenic, as *β*2GP1 is not required to bind cardiolipin. However, multiple studies have provided evidence of thrombosis being associated with *β*2GP1-independent aPL, suggesting a pathogenic role. Nevertheless, evidence of *β*2GP1-dependent aPL being associated with CMV has been shown [16, 17]. It is possible that patients can produce various types of antibodies, not necessarily always associated with increased risk of blood clots. In one case, a patient’s lymphocytes were studied *in vitro* and were induced into pathogenic or nonpathogenic lines by being exposed to the Epstein–Barr virus [25]. These data suggest that the mechanisms between pathogenic and nonpathogenic antibodies are not rigid and that certain individuals can produce either type of antibody. In our case, we found that a prolonged aPTT that did not correct with a mixing study was evidence for lupus anti-coagulant and positive anti-cardiolipin IgM and anti-phosphatidylserine IgM, but negative anti-*β*2 glycoprotien antibodies. Establishing a diagnosis of APS requires meeting both clinical and laboratory criteria.

Based on the revised classification criteria for the APS, patients must meet the following clinical criteria: (1) vascular thrombosis: at least one episode, can occur in any organ or tissue, venous or arterial; and/or (2) pregnancy morbidity: one or more episodes of foetal loss beyond 10 weeks with normal morphology or one or more premature births with normal morphology before 34 weeks due to eclampsia/severe eclampsia/placental insufficiency or three or more consecutive spontaneous abortions before 10 weeks with any anatomic or chromosomal aetiologies excluded [1, 18, 26]. Furthermore, patients must meet the following laboratory criteria: (1) anticardiolipin antibody of IgG and/or IgM in serum or plasma in medium or high titre (>40 GPL or MPL on two or more occasions at least 12 weeks apart), measured by ELISA; (2) lupus anticoagulant present in serum on two or more occasions at least 12 weeks apart; (3) anti-*β*2 glycoprotein-I antibody of IgG or IgM in serum or plasma, present on two or more occasions at least 12 weeks apart, measured by ELISA. Definitive APS is present if at least one clinical criterion is met and at least one laboratory criterion is met with the second measurement occurring at least 12 weeks after symptoms occur [1, 18, 26].

In our case, the patient had no prior history of thrombophilic risk factors, acquired or genetic. Our study points to CMV being a significant aetiology for primary thrombosis in immunocompetent individuals. Multiple splenic infarcts on imaging and abnormal laboratory values prompted the medical team to investigate a viral aetiology. Subsequently, a diagnosis of primary CMV infection with APS was made due to confirmatory serology for high-titre anti-cardiolipin IgM (Table 1). In this patient, an important test that was also positive was the anti-phosphatidylserine antibodies test. These markers are becoming recognized as an important tool for diagnosing antiphospholipid antibody syndrome, especially when anti-cardiolipin and anti-*β*2 glycoprotein antibodies are negative or low [27–30]. Given the ultimate normalization of antiphospholipid antibodies in this case, it is not clear whether she has definite antiphospholipid antibody syndrome – see the discussion of criteria above – versus a self-limited thrombophilic state due to primary CMV together with positive antiphospholipid antibodies.

Multiple studies have elucidated the procoagulant properties of CMV, which has the potential for transient thrombosis in primary infections [7–15]. There have also been a limited number of prior case reports connecting CMV infection with APS [16, 17]. This raises the concern that when treating primary CMV, it may be necessary to screen for thrombotic risk factors and when treating a patient for thrombosis it is necessary to maintain a high degree of suspicion for CMV as the causal factor. Due to the suspected transient nature of the infection, which was confirmed with declining titres of CMV, the patient was placed on anti-coagulation for a limited period of time, and ultimately the anti-cardiolipin and APSA antibody titres came down. There is no specific evidence to guide the length of treatment for APS associated with CMV, but there is the possibility that the recurrent risk of thrombosis is reduced with the clearance of detectable CMV and the reduction of APS antibody titres. Although there is no clear consensus, the ideal management and treatment of APS must be tailored to the patient based on their history of thrombosis and clinical comorbidities. In conclusion, our report bears evidence that CMV can be a potentially significant cause of APS-induced thrombosis. Further studies are needed to understand the mechanisms behind CMV-induced APS and the development of preventative treatments. Based on our report and the evidence provided in other case reports, it is clear that physicians need to be educated regarding the potential relationship between thrombosis, APS and primary CMV infection. 
